# Nutrient Enrichment Predominantly Affects Low Diversity Microbiomes in a Marine Trophic Symbiosis between Algal Farming Fish and Corals

**DOI:** 10.3390/microorganisms9091873

**Published:** 2021-09-03

**Authors:** Adriana Messyasz, Rebecca L. Maher, Sonora S. Meiling, Rebecca Vega Thurber

**Affiliations:** 1 Department of Microbiology, Oregon State University, Corvallis, OR 97331, USA; beccaluciamaher@gmail.com (R.L.M.); rvegathurber@gmail.com (R.V.T.); 2 Environmental Sciences Graduate Program, Oregon State University, Corvallis, OR 97331, USA; 3 Institute of Ecology and Evolution, University of Oregon, Eugene, OR 97403, USA; 4 Center for Marine and Environmental Studies, University of the Virgin Islands, St. Thomas 00802, U.S. Virgin Islands; sonora.meiling@gmail.com

**Keywords:** coral reefs, *Stegastes nigricans*, turf algae, 16S, nutrient pollution, marine bacteria, microbial symbiosis

## Abstract

While studies show that nutrient pollution shifts reef trophic interactions between fish, macroalgae, and corals, we know less about how the microbiomes associated with these organisms react to such disturbances. To investigate how microbiome dynamics are affected during nutrient pollution, we exposed replicate *Porites lobata* corals colonized by the fish *Stegastes nigricans*, which farm an algal matrix on the coral, to a pulse of nutrient enrichment over a two-month period and examined the microbiome of each partner using 16S amplicon analysis. We found 51 amplicon sequence variants (ASVs) shared among the three hosts. Coral microbiomes had the lowest diversity with over 98% of the microbiome dominated by a single genus, *Endozoicomonas*. Fish and algal matrix microbiomes were ~20 to 70× more diverse and had higher evenness compared to the corals. The addition of nutrients significantly increased species richness and community variability between samples of coral microbiomes but not the fish or algal matrix microbiomes, demonstrating that coral microbiomes are less resistant to nutrient pollution than their trophic partners. Furthermore, the 51 common ASVs within the 3 hosts indicate microbes that may be shared or transmitted between these closely associated organisms, including *Vibrionaceae* bacteria, many of which can be pathogenic to corals.

## 1. Introduction

The role of bottom-up forcing [1] on trophic interactions and nutrient-dependent symbioses is profound, particularly in ecosystems that are oligotrophic, such as coral reefs. It is well established that nutrient enrichment can increase macroalgal growth in the absence of fish herbivory, resulting in corals becoming overgrown, shaded, and/or diseased [2,3,4,5]. These combined negative effects on coral physiological traits (inhibition of coral recruitment and growth) can shift a reef from a coral-dominated to algal-dominated state which makes corals less resilient to disturbances such as bleaching, disease, and hurricanes [5,6,7,8,9]. 

In this study, we examine coral-associated algal farming fish in the genus *Stegastes*. These fish farms alter algal assemblages on coral by removing fleshy macroalgae and cultivating filamentous turf algae, which the fish find more palatable [10,11]. *Stegastes* defend these food resources by demonstrating aggression towards other reef fish such as corallivores, herbivores, and egg predators, while ignoring most carnivores and omnivores [12]. *Stegastes* territories are dominated by the epilithic algal matrix (EAM), a conglomeration of turf algae, juvenile macroalgae, detritus, invertebrates, and bacterial assemblages [13,14]. Previous studies have documented numerous detrimental effects of EAM on corals including inhibition of coral recruitment [15,16], decreases in coral growth [17,18,19], and coral physiological stress [20,21,22]. Further, evidence suggests that territorial *Stegastes* farming may promote the development of reservoirs of potential coral disease pathogens (genera *Geitlerinema, Leptolyngbya, Oscillatoria*, and *Sphingomonas*) within the EAM [23] and within *Stegastes* territories (genera *Leptolyngbya* and *Oscillatoria*) [10]. Additionally, the presence of territorial *Stegastes nigricans* increases the rate of coral mortality [23] and may further increase algal growth by enhancing nutrient supply via recycled waste products [24]. While we understand many macro-scale aspects of the trophic relationship between corals, fish, and turf algae under nutrient enrichment, we know much less about the interactions and dynamics among the microbiomes of these hosts under nutrient enrichment. 

Studying multiple hosts at the microbial scale gives new insights into the interactions between coral reef organisms. Microbial sampling of the coral-algal interface uncovered unique microbial assemblages characterized by higher microbial abundances and larger microbial cells in the interface compared to the coral or the turf algae [25]. We also previously showed that fishes have the capability to share and transfer microbiome members with corals. This includes the potential coral pathogens *Vibrio vulnificus* and *Photobacterium rosenbergii* which significantly changed in abundance in corals exposed to Surgeonfish feces and thermal stress [26]. Parrotfish predation on corals also resulted in increased bacterial alpha diversity and the detection of both beneficial and opportunistic bacteria was only found on corals post predation, indicating direct transmission of bacteria from fish mouth to coral mucus and tissue, or indirectly facilitating bacterial growth or invasion within the coral or from the surrounding environment [27]. 

At the same time, nutrient pollution in oligotrophic coral reef ecosystems can alter the structure and function of coral holobionts (host, symbiont, microbiome, and virome), mediating changes in the symbiotic interactions among its members [28,29]. Corals rely on their symbiotic dinoflagellate partners, family Symbiodiniaceae, for carbon production [30,31,32] and the breakdown of this symbiosis is known as coral bleaching, which can lead to coral starvation and death [33,34]. Research on inorganic nutrient amendment has revealed the depletion of phosphate can stress coral Symbiodiniaceae and lead to bleaching [35]. Additionally, nitrogen addition can counteract the nitrogen limitation necessary for a stable coral-algal symbiosis and can also lead to bleaching [36,37]. Corals also rely on their bacterial partners, or their microbiomes, to produce antimicrobial compounds that can defend the coral from pathogens [38], and nutrient cycling including carbon, nitrogen, sulfur, and phosphorous [39,40]. The combined effects of overfishing and nutrient enrichment are known to destabilize coral microbiomes and increase putative pathogen loads [29]. Coral microbiomes undergoing stressors such as nutrient enrichment demonstrate higher microbiome variability, particularly when exposed to nitrate or ammonia [41] or the combination of nitrogen and phosphorus [42,43]. In addition, the combination of stressors such as nutrients, predation, and increased temperatures have an antagonistic effect on microbial diversity [44]. 

Ultimately, shifts in the abundance of limiting nutrients can impact both the trophic interactions among corals, fish, and algae and their microbiomes in ways that negatively impact coral health, resilience, and recovery [45,46,47]. Yet, while much research has focused on the effects of nutrients on the benthic community and coral microbiome dynamics, few, if any, have explored how nutrient pollution might affect microbial interactions among these members of the benthic community. Additionally, it is unclear how the destabilization of the coral microbiome under nutrient enrichment compares to the microbiomes of other hosts in close association with corals. These microbiome comparisons can help us understand whether corals are particularly susceptible or robust to nutrient pollution. 

To test how nutrient enrichment might alter microbiome interactions among members of an ecological symbiosis, we compared *Porites lobata* coral microbiomes to the microbiomes of their resident fishes, *Stegastes nigricans*, and their farmed EAM gardens of turf algae or the ‘algal matrix’. This in situ 8-week nutrient pulse experiment allowed us (1) to test which microbes are shared among or are unique to the 3 partners, (2) to identify microbes that are variably sensitive to nutrient amendment, and (3) to compare microbiome resistance and resilience across the 3 different hosts that represent unique trophic levels. We hypothesized that coral microbiomes would be uniquely susceptible to nutrient pollution because of their low microbiome diversity [48] while fish and turf algae would be more resistant due to their high microbiome diversity [49,50,51]. Additionally, we hypothesized that fish and algal matrix microbiomes would reflect similar changes during the experiment because of the close trophic symbiosis between the two (e.g., algal farming and consumption by the fish). We also hypothesized that microbiomes would generally increase in diversity and become more variable under nutrient enrichment by supporting bacterial communities with high variability and increased presence and abundance of opportunistic bacterial taxa. 

## 2. Materials and Methods

### 2.1. Experimental Setup and Sampling

**Figure 1:**
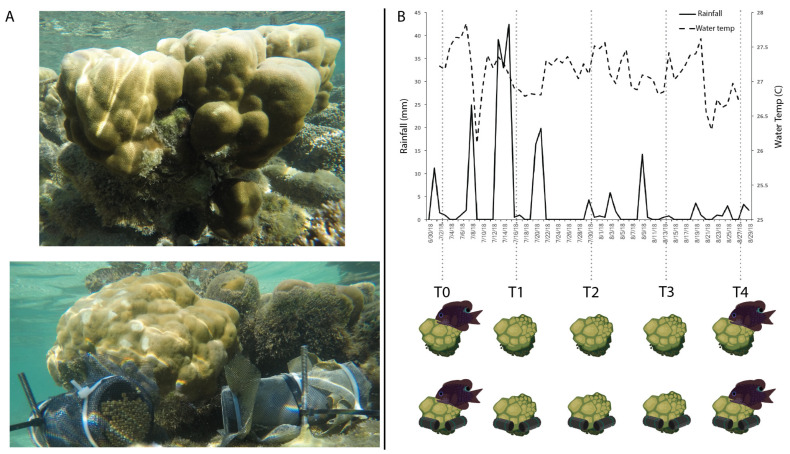
Experimental nutrient enrichment set up. (**A**) In situ *Porites lobata* colonies inhabited by turf algae farming *Stegastes nigricans*, on a northern fringing reef in Mo’orea French Polynesia, were left untreated (*n* = 7) (top image), or exposed to Osmocote © slow-release nutrient diffusers (*n* = 7) (bottom image). (**B**) Sampling of *P. lobata*, algal matrix, and *S. nigricans* started on 7/02/18, and continued over an 8-week period, sampling every two weeks. Local rainfall and water temperature was recorded over the experimental period (B top graph). The fish *S. nigricans* were only sampled on the first and last timepoints (T0 and T4), while the coral *P. lobata* and turf algae were sampled for every timepoint (T0 to T4) (B bottom images).

*Porites lobata* colonies inhabited by *Stegastes nigricans* fish that were actively farming turf algae were used for this experiment. Colonies were randomly selected along Maharepa reef, in Mo’orea, French Polynesia (17.483194 S, 149.814056 W). Control colonies (*n* = 7) were left undisturbed and nutrient treated colonies (*n* = 7) were exposed to slow-release nutrient diffusers with 200 g Osmocote © classic (19-6-12 N-P-K) in 15.25 cm PVC pipes covered in mesh (Figure 1A). Sampling for both treatments began (Time 0 or T0) following nutrient diffuser installation. Coral mucus, tissue, and skeleton were sampled with bonecutters, and stored in 6 mL DNA/RNA Shield (Zymo Research, Irvine, CA, USA) with sterilized matrix A (MP Biomedicals, Santa Ana, CA, USA). Turf algae or the ‘algal matrix’, was sampled with different bonecutters and stored in 2 mL Zymo DNA/RNA shield lysing tubes. Coral and algal matrix samples were taken for all timepoints. We could only obtain fish samples for the first (T0) and last (T4) timepoints because most colonies were inhabited by ~3–5 fish; as sampling required sacrificing the fish, if fish were sampled at each timepoint the original cohort would have been removed and a new cohort that would not have undergone the entire pulse treatment would have inhabited the colony. 

For T0, *S. nigricans* were caught via spear-gun, and for T4 they were anesthetized with a 1:5 clove oil to ethanol solution (Jedwards International, Inc. Braintree, MA, USA) and collected with hand nets. For both time-points *S. nigricans* were euthanized on the reef in whirl-paks with MS222 (100 mg of MS222 into 0.5 L seawater, balanced to a pH of ~7.5 w/500 mg NaCO_3_) and kept on ice until dissected (~1 h). In the lab, the *S. nigricans* were dissected with a sterile scalpel, and organ pieces (kidney, liver, gut) were stored in 2 mL Zymo DNA/RNA shield lysing tubes. All *S. nigricans* samples were collected under approval of the Institutional Animal Care and Use Committee (IACUC) at Oregon State University (Animal Care and Use Protocol #5056). All host samples stored in DNA/RNA Shield tubes were bead-beaten for 20 min before aliquots of host tissue slurry were taken for DNA extraction.

### 2.2. DNA Extraction, 16S Library Preparation, Sequencing

To extract DNA from samples from each host, 250 μL of slurry preserved in DNA/RNA Shield (Zymo) was input into the Qiagen DNeasy Power-Soil kit and DNA was extracted according to the kit protocol. Next, the V4 region of the 16S rRNA gene was amplified via 2-step PCR coupling forward and reverse primers 515F (5′-GTG YCA GCM GCC GCG GTA A-3′) [52] and 806R (5′-GGA CTA CNV GGG TWT CTA AT-3′) [53]. First-step PCR was conducted according to the reaction and thermocycler protocol described in Maher et al. 2020 [54]. Second-step PCR was conducted according to the methods described in Ezzat et al. 2021 [26]. Briefly, each 16S band visualized on a 1.5% agarose gel was poked with a pipette tip and swirled into a second-step barcoding master mix solution which was then run on a thermocycler for barcoding. Lastly, amplicons were pooled into equivolume ratios in a single pool and cleaned using Agencourt^®^ AMPure XP beads. Libraries were then sequenced at Oregon State University (OSU) by the Center for Quantitative Life Sciences (CQLS) with v.3 reagent 2 × 300 bp read chemistry on an Illumina MiSeq.

### 2.3. Data Processing

Demultiplexed reads from the CQLS were trimmed of primers, adapters, and barcodes using Cutadapt (v 3.1). Reads were then processed separately for each host using DADA2 (v 1.16.0) [55] in R (v 4.0.0) [56]. Forward and reverse reads were truncated at their 3’ end at 260 and 210 base pairs, respectively. Sequences were truncated at the first position having a quality score less than or equal to 2, and reads with a total expected error >2 or with the presence of Ns were discarded. Error rates were then learned independently on filtered forward and reverse reads, followed by dereplication and sample inference. Next, forward and reverse reads were merged and an amplicon sequence table was constructed. Two-parent chimeras (bimeras) were removed and taxonomy was assigned at 100% sequence identity using the Silva reference database (v132) [57]. The resulting unique ASVs for each host were imported into phyloseq (v 1.32.0) [58]. ASVs that were annotated as mitochondrial or chloroplast sequences as well as ASVs with a Kingdom classification of “NA” were removed. Two algae samples were determined to have insufficient sequencing depth (<5000 reads) based on the distribution of sequencing depths for algae samples (Figure S1). The removal of these algae samples did not impact the total number of microbial taxa. ASVs were then agglomerated to a genus level classification via phylogenetic distance (tip_glom, h = 0.05) and rarefied to the lowest sequence number for each host (Coral: 2308, Fish: 10,097, Algae: 5973). Changes in read numbers per sample throughout data processing are recorded in the Supplemental Files (Coral: File S1, Algae: File S2, Fish: File S3). Changes in the number of taxa for each host throughout phyloseq filtering is outlined in Table S1. A phylogenetic tree was made from the resulting ASVs for each host using QIIME 2 2019.10 [59]. Briefly, ASVs were aligned with mafft [60] via the q2-alignment plugin and used to construct a phylogeny with fasttree2 [61] via the q2-phylogeny plugin.

### 2.4. Statistical Analyses

Alpha diversity metrics including observed richness and Simpson’s Diversity index and beta diversity statistics were run separately for each host. To improve the normality of observed richness for each host, observed richness was square-root transformed. Simpson’s index was arcsine-transformed to improve normality for coral samples, while transformation of this metric was not necessary for fish or algae samples. Experimental group effects on each alpha diversity metric were assessed with linear mixed effect models (LMM) using lme4 (v.1.1.23) [62] with time, treatment, and their interaction as fixed effects and individual coral colony as a random effect. Multiple comparisons were performed with estimated marginal means (EMMs) using the emmeans (v.1.4.8) package. For beta diversity statistics, Bray-Curtis dissimilarities were first calculated in phyloseq. Next, Permutational Multivariate Analyses of Variance (PERMANOVA) [63] were conducted to test for differences in bacterial community compositions between treatment groups or across time and between group factorial interactions (Treatment*Time). In addition, Permutational Analyses of Multivariate Dispersions (PERMDISP) [64] were used to test for homogeneity of multivariate dispersions between groups and to calculate the distance to centroid for each sampling group. PERMANOVA and PERMDISP were performed using the functions adonis and betadisper in the package vegan (v.2.5.6) followed by a pairwise analysis of variance with the pairwise.adonis.dm function and permutest in vegan, respectively, with FDR adjusted *p*-values.

Additionally, changes in the abundance of different bacterial genera across time and treatment for each host were assessed with Analysis of Composition of Microbiomes (ANCOM) with controls for false discovery rate [65]. For each host, an unrarefied ASV table agglomerated to the genus-level (as described above) was used as input into ANCOM. Fish and algae samples were further filtered to only include ASVs with at least 3 counts in 20% of the samples. For each host, ANCOM was run with a model including time, treatment, and their interaction as fixed effects and the individual coral colony as a random effect. A significance level of W = 0.7 was used in which the null hypothesis for a given taxon was rejected in 70% of the tests and p-values were corrected with Benjamini-Hochberg FDR [65,66]. 

### 2.5. Shared ASVs

The ASV phyloseq object prior to genus agglomeration, was rarefied to the minimum number of sequences for each host (coral: 2308, fish: 10,097, algae: 5973). Shared ASV’s were found via the Reduce and intersect functions in base R. The relative abundance of the shared ASV’s was calculated via the transform function in the microbiome (v.1.10.0) package and visualized via the plot_heatmap function in phyloseq (v.1.32.0).

### 2.6. Water Nutrient Analysis

Water samples for nutrient analysis were taken at time-points T0, T2, and T4 from 6 random colonies (*n* = 3 for each treatment). Water was collected in whirl-paks and stored on ice until filtered (5 mL through 0.2 um). Precipitation and water temperature data was accessed via the Mo’orea Coral Reef LTER data repository [67,68]. At the CEOAS Chemical Analysis Lab (OSU) the continuous segmented flow systems were utilized to determine inorganic nutrients in seawater. Technicon AutoAnalyzer II™ components were used to measure phosphate [69]; and Alpkem RFA 300™ components were used for nitrate plus nitrite and nitrite [70,71]. A detailed description of the continuous segmented flow procedures used can be found in Gordon et al. (1994) [72].

## 3. Results

### 3.1. Environmental Conditions Significantly Varied over 8-Week Experimental Period

Throughout the experimental time period, coral health and algal matrix growth were visually monitored during each sampling timepoint. No significant changes in phenotype were observed throughout the experiment for both control and nutrient treated coral colonies. Environmental conditions, such as water temperature and rainfall in the area were recorded, with the greatest increase in rainfall and decrease in temperature occurring between T0 and T1 (Figure 1B). Later timepoints (T2–T4) had steadier changes in temperature and rainfall. Changes in concentration (micromole/liter) of measured phosphate (PO_4_), nitrate and nitrite (N + N), and nitrite (NO_2_) over the three sampled time-points (T0, T2, and T4) are plotted in Figure S2. For each nutrient, no significant changes occurred over treatment or time (Kruskal-Wallis rank sum test, Nutrient ~ Treatment, Nutrient ~ Time). 

### 3.2. Corals Have Increased Sensitivity to Nutrient Enrichment Compared to Their Resident Fish and Algal Matrix

**Figure 2:**
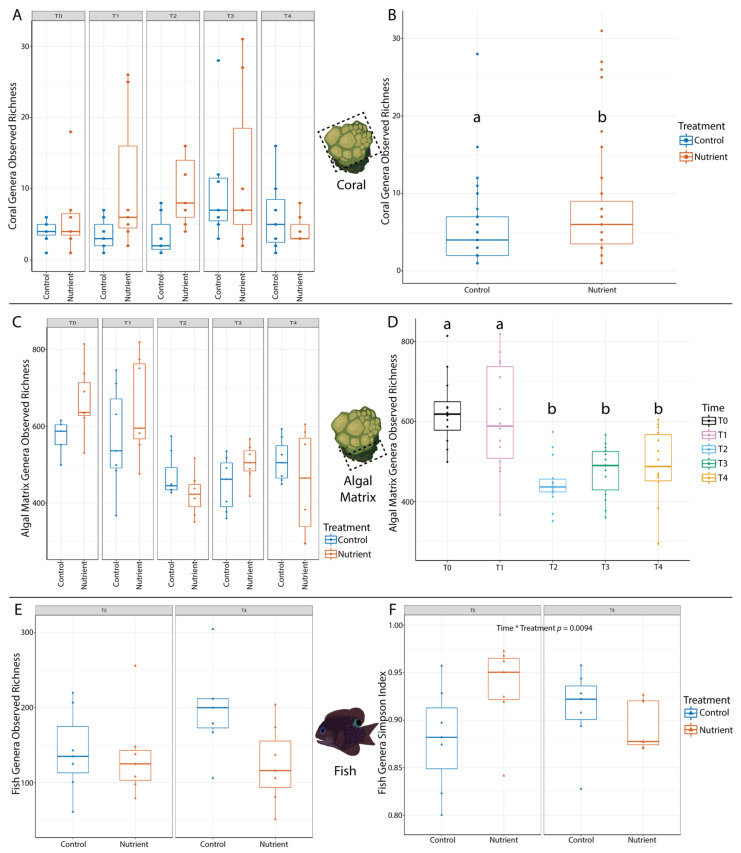
Alpha diversity results for coral, fish, and algal matrix microbiomes. Only statistically significant results are shown in graphs on the right (**B**,**D**,**F**). (**A**) Coral microbiome observed richness increases for nutrient treated colonies over time (T0–T2), and then becomes more similar between treatments from timepoint T3–T4. (**B**) Only treatment had a significant effect on coral microbiome observed richness, with increased diversity for nutrient treated colonies. (**C**) Algal matrix microbiome observed richness changed more over time than between treatments. (**D**) Only time had a significant effect on algal matrix observed richness, with diversity decreasing over time. (**E**) Fish microbiome observed richness increased over time for control colonies and remained similar for nutrient treated colonies. (**F**) Only the Time*Treatment interaction had a significant effect on fish microbiome Simpson diversity. Lower-case letters indicate statistical significance, i.e. boxes that do not have any letters in common are significantly different from one another (**B**,**D**).

Although control corals were left undisturbed, observed species richness changed across the course of the experiment with a transient increase at T3 (6 weeks), which disappeared 2 weeks later at T4 (Figure 2A, blue bars). For nutrient treated corals, within observed species richness increased until T2, stayed similar at T3, and then decreased between T3 and T4 (Figure 2A, red bars). When examined over the whole experiment, nutrient treatment showed significantly increased coral microbial diversity (*p* = 0.045, Fstat = 4.99) (Figure 2B).

In contrast, observed species richness for algal matrix microbiomes decreased over time, with the largest decrease occurring between T1 and T2 (Figure 2C). Algal matrix microbiomes changed more across timepoints than across treatment (Figure 2C) resulting in only ‘time’ having a significant effect on algal matrix microbiome diversity (*p* = 3.72 × 10^−6^ Fstat = 10.53) (Figure 2D). Additionally, while within sample diversity (observed and Simpson) increased for control fish microbiomes over time, there was a decrease in diversity for nutrient treated fish microbiomes across time (Figure 2E,F). The treatment*time interaction had the only significant effect on within sample Simpson diversity of fish microbiomes (*p* = 0.0094, Fstat = 9.52) (Figure 2F). Alpha-diversity statistics for all hosts across all variables tested are recorded in Table S2.

### 3.3. Between Sample Diversity Changed Significantly for Each Host

**Figure 3:**
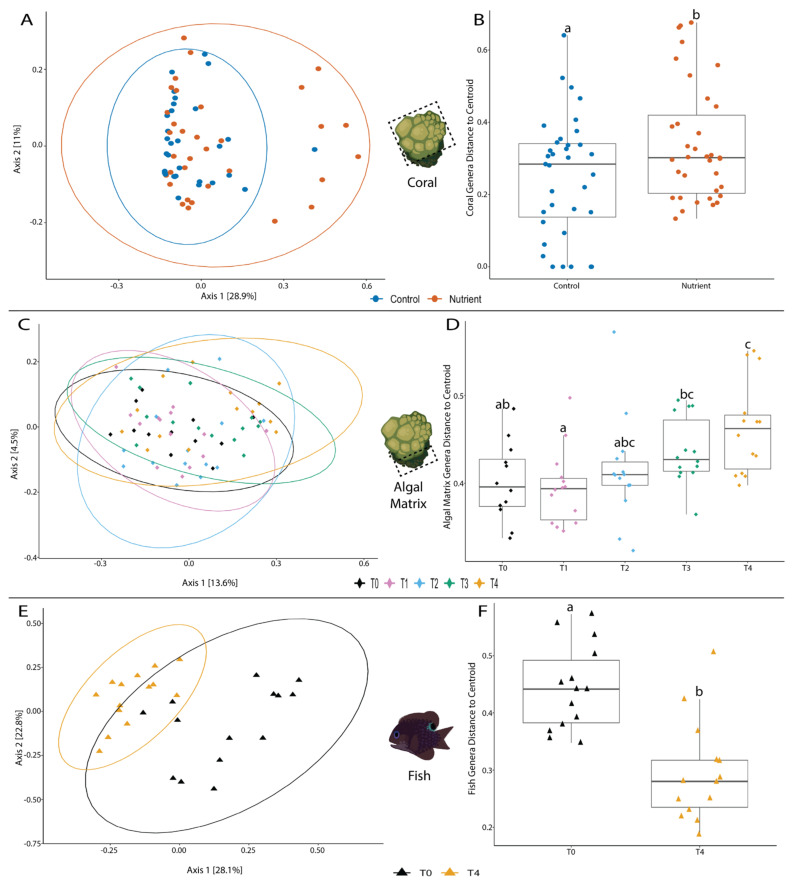
Significant beta diversity results for coral, fish, and algal matrix microbiomes. Bray-Curtis dissimilarity was significantly different between control and nutrient treated coral microbiomes (**A**), and was significantly different across time for algal matrix microbiomes ((**C**), T0–T4) and fish microbiomes ((**E**), T0 and T4). Dispersion/variability, or distance to centroid, was significantly increased for coral microbiomes under nutrient enrichment compared to coral microbiomes under control conditions (**B**). Dispersion significantly increased over time for algal matrix microbiomes (**D**) and decreased over time for fish microbiomes (**F**). Lower-case letters indicate statistical significance, i.e. boxes that do not have any letters in common are significantly different from one another (**B**,**D**,**F**).

We analyzed beta-diversity in two ways: (1) a shift in between sample diversity or the community structure between samples (PERMANOVA) and (2) the dispersion or variability of between sample diversity (PERMDISP; how dissimilar samples were from one another). Coral microbiome between sample diversity was significantly affected by nutrients, while only time had a significant effect on the between sample diversity of algal matrix and fish microbiomes (Figure 3). Between sample diversity was significantly different for control and nutrient treated coral microbiomes (*p* = 0.006**, R2 = 0.041) (Figure 3A) and dispersion significantly increased for nutrient treated coral microbiomes (*p* = 0.023*) (Figure 3B). In other words, coral microbiomes became more dissimilar from one another after nutrient exposure. In contrast, between sample diversity significantly changed over time for algal matrix microbiomes (*p* = 0.006**, R2 = 0.079) (Figure 3C) and dispersion significantly decreased for algal matrix microbiomes over time (*p* = 0.0027**) (Figure 3D). Like algal matrix microbiomes, between sample diversity was significantly different for fish microbiomes over time (*p* = 0.001***, R2 = 0.21) (Figure 3E), and dispersion significantly decreased from T0 to T4 (*p* = 4.93 × 10^−5^***) (Figure 3F). The treatment*time interaction had a significant effect on algal matrix microbiome dispersion (*p* = 0.0068) as well as the between sample diversity (*p* = 0.025*, R2 = 0.069) and dispersion/variability (*p* = 1.76 × 10^−5^) of fish microbiomes. Beta-diversity statistics for all hosts across all variables tested are recorded in Tables S3 and S4.

### 3.4. Coral Microbiomes Are Dominated by One Bacterial Genus While Fish and Algae Microbiomes Are More Even

**Figure 4:**
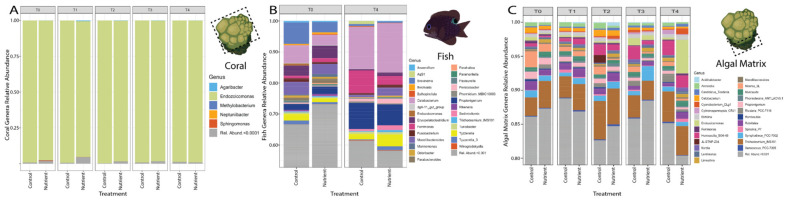
Relative abundance of bacterial genera across the three host microbiomes. (**A**) Over 90–99% of coral microbiomes were dominated by one genera *Endozoicomonas*. (**B**) Approximately 60–70% of fish microbiomes were dominated by genera with a relative abundance of less than 0.001 and changes in taxa are more apparent across time than across treatment. (**C**) Approximately 80–85% of algal matrix microbiome genera had a relative abundance of less than 0.001 and again, changes in taxa are more apparent across time than across treatment. The range of the *y*-axis of graphs (**B**,**C**) have been altered to highlight the more abundant taxa.

Each host had a unique microbial community (Figure S3), characterized by dominant or minor taxa. Coral microbiomes, across time and treatment, were dominated by the *Endozoicomonas* bacterial genus, which made up over 90–99% of every sample (Figure 4A). In contrast, both fish and algal matrix microbiomes were dominated by minor taxa. For fish microbiomes (1095 total taxa), ~70% of taxa in T0 had a relative abundance of less than 0.001, and ~60% of taxa in T4 had a relative abundance of less than 0.001 (Figure 4B). Some of the more abundant taxa (rel. abund. > 0.001) in fish T0 microbiomes include *Brevinema, Cetobacterium, Erysipelatoclostridium,* and *Macellibacteroides*, and for fish T4 microbiomes include *Cetobacterium, Ferrimonas, Propionigenium,* and *Tyzzerella.* Across time and treatment, ~80–85% of taxa in algal matrix microbiomes (6179 total taxa) had a relative abundance of less than 0.001 (Figure 4C). Some more abundant (rel. abund. > 0.001) algal matrix microbiome taxa include *Hormoscilla_SI04-45*, *Moorea_3L*, *Propionigenium*, *Symphothece_PCC-7002*, and *Trichodesmium_IMS101*.

### 3.5. Fish and Algae Microbiomes Had Several Taxa Significantly Varying in Abundance over Time

**Figure 5:**
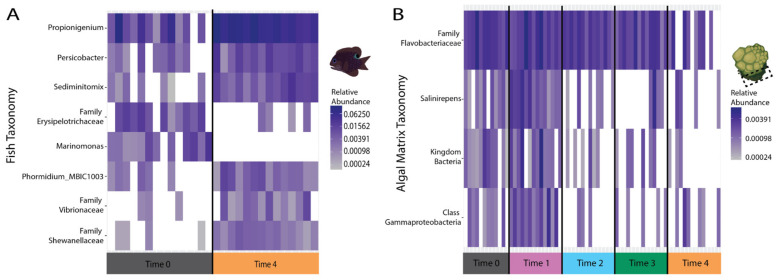
Fish and algal matrix bacterial taxa with significant changes in abundance. (**A**) Seven bacterial taxa (*Propionigenium, Persicobacter, Sediminitomix, Marinomonas, Phormidium_MBIC10003,* Family *Vibrionaceae,* and Family *Shewanellaceae*) within fish microbiomes changed significantly over time and one taxa (Family *Erysipelotrichaceae*) changed significantly over time and the treatment*time interaction. (**B**) Four bacterial taxa (Family *Flavobacteriaceae, Salinirepens,* Kingdom *Bacteria,* and Class *Gammaproteobacteria*) within algal matrix microbiomes changed significantly over time and the treatment*time interaction. Coral microbiomes did not have any taxa that significantly changed over treatment, time, or the interaction of these variables. If not identified to genus, the next higher classification taxonomic assignment is used. Darker colors indicate higher abundance, and no color indicates an absence of that taxa.

For fish microbiomes, 8 bacterial genera (named to the lowest identifiable taxonomy) showed significant changes (ANCOM detection = 0.7) in relative abundance over time (Figure 5A) and all of these, with the exception of the Family *Erysipelotrichaceae*, were significant for both time and the treatment*time interaction. Family *Erysipelotrichaceae* and *Marinomonas* both decreased in abundance over time. *Propionigenium*, *Persicobacter*, *Sediminitomix*, *Phormidium_MBIC10003*, Family *Vibrionaceae* (BLAST nr similarities to different *Vibro* species, Table S5), and Family *Shewanellaceae* (BLAST nr similarity to *Paraferrimonas haliotis*, which is in Family *Ferrimonadaceae*) increased in abundance over time. For fish microbiomes, the taxa identified to genera with significant differential abundance across time (*Marinomonas*, *Propionigenium*, *Persicobacter*, *Sediminitomix*, *Phormidium_MBIC10003*) (Figure 5A) were all part of the more abundant genera (abundance >0.001) (Figure 4B).

For algal matrix microbiomes, 4 genera had significant changes (ANCOM detection = 0.7) in relative abundance over time (Figure 5B), and time and the treatment*time interaction had a significant effect on all of these genera (named to the lowest identifiable taxonomy): Family *Flavobacteriaceae, Salinirepens,* Kingdom *Bacteria,* and Class *Gammaproteobacteria*. Family *Flavobacteriaceae* decreased in abundance from T3 to T4. *Salinirepens,* Kingdom *Bacteria* (BLAST nr similarity to uncultured Phylum *Planctomycete* and uncultured Class *Gammaproteobacteria*), and Class *Gammaproteobacteria* (one BLAST nr similarity to *Saccharophagus degradans*), all increased from T0 to T1, decreased from T1 to T2 and stayed similar to T4. For algal matrix microbiomes, the one taxon identified to genus with significant differential abundance across time (*Salinirepens*) is part of the minor taxa (abundance <0.001). No genera with significant changes in relative abundance were detected for coral microbiomes.

### 3.6. Despite a Strong Trophic Interaction Few Microbes Are Shared among the Three Hosts

**Figure 6:**
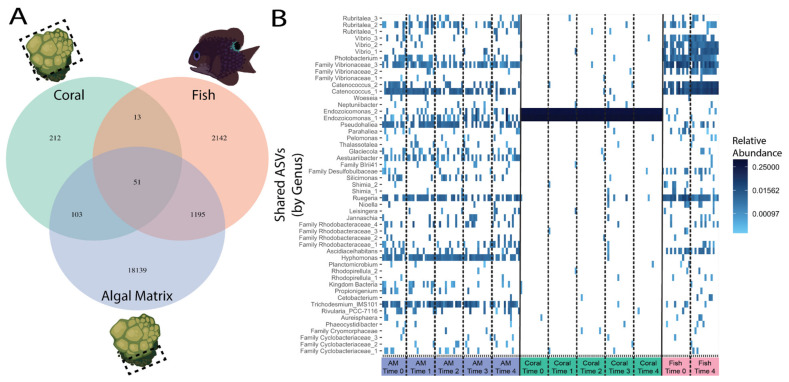
Unique and common ASVs across the three host microbiomes. (**A**) Venn-diagram of unique and shared ASVs between the 3 hosts shows that all hosts had 51 ASVs in common. (**B**) Relative abundance of these 51 ASVs for each host across time (‘AM’ = algal matrix). Darker colors indicate higher abundance, and no color indicates an absence of that taxa.

All hosts shared 51 ASVs in common. Fish microbiomes had ~10X and algal matrix microbiomes had ~100× more ASVs than coral microbiomes (Figure 6A). Fish microbiomes shared more ASVs with algal matrix microbiomes than coral microbiomes (Figure 6A). Coral microbiomes shared more ASVs with algal microbiomes than fish microbiomes (Figure 6A). The 51 shared ASVs changed in relative abundance over time, depending on host (Figure 6B). The *Endozoicomonas* ASVs shared between the 3 hosts were the most dominant out of all 30 *Endozoicomonas* ASVs found in coral microbiomes (Figure S4). Apart from these dominant *Endozoicomonas*, all other 49 shared ASVs were generally present in the corals in low abundance, and most increased in abundance at T3 but then decreased at T4. Several shared ASVs were more highly abundant in both fish and algal matrix microbiomes: *Rubritalea* (_2), Family *Vibrionacae* (_3), *Catenococcus* (_1), and *Ruegeria*. ASVs that were highly abundant in only algal matrix microbiomes include: *Pseudohaliea*, *Aestuariibacter*, *Hyphomonas*, and *Trichodesmium_IMS101*. ASVs that were highly abundant in only fish microbiomes include: 3 *Vibrio* ASVs, *Catenocccus* (_1), and *Ascidiaceihabitans*. BLAST nr similarities: Kingdom Bacteria ASV to uncultured bacterium, Family Vibrionaceae_2 ASV to *Vibrio sagamiensis*, Family Cryomorphaceae ASV to uncultured *Owenweeksia* sp., Family Cyclobacteriaceae_1 and Family Cyclobacteriaceae_3 ASVs to Family *Flammeovirgaceae*, Family Rhodobacteraceae_3 to *Epibacterium* sp., Family Rhodobacteraceae_4 to *Pseudoruegeria lutimaris* and *Maritimibacter alkaliphilus*.

## 4. Discussion

Anthropogenic sources of nutrients contribute to coral reef decline [45,46]. Numerous studies have looked at how nutrient pollution in oligotrophic coral reef ecosystems alters both coral-fish-algae trophic dynamics and the coral holobiont (host, symbiont, microbiome, and virome) structure and function [11,28,29]. For example, previous studies show that *Stegastes* exclusion and nutrient enrichment increase turf algae cover and alter trophic dynamics in reef ecosystems [11], but the interactions among the microbiomes of the corals, fish, and algae under such experiments are yet to be researched. It is well-established that coral disease, such as tissue loss and decay, is correlated with the destabilization of mutualistic coral microbial communities [73]. However, it was recently shown that overfishing and nutrient pollution surprisingly interact to increase the susceptibility of corals to mortality from typically unharmful fish predation [28,29,47]. Given this link to nutrient enrichment and increased disease and mortality from fish predation, we posited that symbioses between algal matrix farming fishes and their coral hosts would be negatively affected by the addition of nitrogen and phosphorus into highly oligotrophic habitats such as those on the island of Mo’orea. To evaluate both which microbes are shared within this unique trophic interaction and determine how nutrients might alter this relationship, we exposed several *Stegastes nigricans* colonized *Porites lobata* corals to nutrient enrichment over 8 weeks and sampled the fishes, farmed algal matrix, and corals at different frequencies.

### 4.1. Coral Microbiomes Respond Uniquely to Nutrient Enrichment

In this study, nutrient enrichment significantly changed the coral microbiome within and between sample diversity, while enrichment on its own did not have a significant impact on the within and between sample diversity of fish and algal matrix microbiomes (Figure 2 and Figure 3). This indicates that coral microbiomes may be less resistant to nutrient enrichment than algal matrix and fish microbiomes. However, changes in coral microbiome observed richness over time and treatment (Figure 2A) also indicates that coral microbiomes may be resilient to nutrient enrichment, as richness levels between treatments became more similar by T3, which would be the end of the nutrient pulse, and stay similar till the last time point (T4). We previously saw similar patterns in coral microbiomes after exposure to a natural temperature anomaly in which species richness peaked after peak temperatures, and declined following a recovery period [54]. 

Coral microbiome dispersion appears to be most significantly impacted by nutrient enrichment as it significantly increased in nutrient treated corals, indicating that coral microbiomes become more dissimilar under nutrient enrichment. This significant increase in dispersion is most likely also driving the significant shift we calculated in the community structure between samples (Figure 3A). When calculating significance in community shifts (PERMANOVA) we assume dispersion is equal across our samples, but since this was not the case, the significant changes in dispersion may be influencing the significant shifts in community structure between samples. There does not appear to be as notable a shift in communities in coral samples between treatments (Figure 3A) and algal matrix samples across time (Figure 3C) as there is in fish samples over time (Figure 3E). Therefore, the statistically significant results we see in coral and algal matrix microbiome shifts may be driven by the significant change in dispersion we find in each host (Figure 3B,D). 

The unique effect of nutrient enrichment on coral microbiomes may have occurred because coral microbiomes are less diverse compared to fish and algal matrix microbiomes. Our coral microbiome samples were dominated by *Endozoicomonas*, a common coral-associated bacteria [74]. There were 30 *Endozoicomonas* ASVs present in coral samples with varying abundances (Figure S4) and the richness of *Endozoicomonas* ASVs changed more over time rather than treatment (Figure S5A) with a significant increase in richness between T1 and T3 (Figure S5B). Therefore, even though *Endozoicomonas* accounted for approximately 98% of the coral microbiome for each sample, the diversity of this genera significantly changed over time and not treatment, so it is most likely not a main driver of the increase in diversity we detected for the whole coral microbiome under nutrient enrichment (Figure 2B). This made us question whether less abundant coral microbial taxa may be driving the changes we see in alpha- and beta-diversity. However, no taxa in coral microbiomes were found to be differentially abundant across treatment, time, or the treatment*time interaction. 

It was difficult to discern what was driving changes in coral diversity between treatments, however, our ability to compare coral microbiomes to fish and algal matrix microbiomes gave us some more insights. Compared to the coral microbiomes, the more even and diverse fish and algal matrix microbiomes may be contributing to their resistance to change under nutrient enrichment. It is still unclear whether microbial richness and evenness contribute to resistance and resilience [75], yet some microbial experiments have showed that higher evenness and richness support functional stability and contribute to resistance and resilience to environmental stress [76,77]. Fish and algal matrix microbiomes are more diverse and dominated by minor taxa with an average abundance <0.001 across identified genera. We detected differentially abundant taxa in fish and algal matrix microbiomes, and time and the treatment*time interaction were the only variables significantly impacting their microbial diversity. In comparison, no differentially abundant taxa were found in coral microbiomes, even though treatment had an effect on coral microbiome alpha-diversity and dispersion. These results give an initial indication that overall microbiome diversity and evenness may play a role in buffering any changes driven by nutrient enrichment. 

### 4.2. Cryptic Environmental and/or Biological Factors May Be Altering Fish and Algal Matrix Microbiomes

While nutrient enrichment appears to have the most significant effect on coral microbiome dispersion, fish and algal matrix microbiomes were most consistently impacted by time and the treatment*time interaction. We hypothesized that fish and algal matrix microbiomes would respond similarly and would become more variable, or increase in dispersion, over the experimental period. While we saw this trend with algal matrix microbiomes, fish microbiome dispersion decreased over time. This indicates that while *Stegastes nigricans* eat the turf algae, their microbiomes do not behave similarly. Most research suggests that *S. nigricans* are exclusively herbivores [78,79] that eat the turf algae they farm, however their gut contents are paradoxically not dominated by turf algae [13,14]. It was suggested that detritus within the algal matrix was the main source of nitrogen within the fish diet, while the algae, invertebrates, and sediment found in algal matrices were underrepresented [14]. This may explain the differences we found in the fish and algal matrix microbiomes. If *S. nigricans* diet benefits more from the detritus within the algal matrix than any other component, their microbiomes will not be as similar to the algal matrix as we would expect. 

Because fish and algal matrix microbiomes did not significantly change in response to nutrient amendment alone, we examined whether the significant changes we see in their microbiomes over time (Figure 2 and Figure 3) correlated with other environmental changes, particularly rainfall, which was measured over the experimental period (Figure 1B). Based on the results of differential abundance (Figure 5) it does not appear that fish and algal matrix changes in taxonomic abundance match these few environmental patterns. Increased rainfall would indicate a decrease in water salinity and a potential decrease in halophilic bacteria. However, microbial taxa identified as *Salinirepens*, a halophilic bacteria, within the algal matrix microbiomes increase at the same time we observed the largest amount of rainfall (T1, Figure S6A). We also see an absence of the halophilic *Marinomonas* genera from fish microbiomes at T4, which was preceded with little rainfall (Figure S6B). Again, the high microbial diversity and evenness we found in fish and algal matrix microbiomes may be buffering or complicating microbial changes due to other uncontrolled environmental variables. In addition, we did not measure other variables such as water compounds other than N and P, amount of light exposure, or hormonal/growth changes in the fish, which could be contributing to the changes in differentially abundant bacteria. Additionally, a caveat of our experiment is our inability to know the composition of and changes within fish microbiomes from T1 to T3, since we could only sample at T0 and T4. Microbial samples at these timepoints would have further clarified how fish samples changed over the entire nutrient pulse experiment.

Another caveat of our experiment is our ability to confirm increased nutrient levels from our water samples. Based on the results of changes in nutrient concentration over time (Figure S2), we cannot confirm that nutrient levels actually increased for nutrient treated coral colonies. Our Osmocote © diffusers were set up in a method similar to Vega Thurber et al., 2014, which confirmed an increase in nutrient levels in the water column, however our method for collecting the water was not the same (we did not use syringes), which could be contributing to our results [47]. We also could have sampled *Turbinaria* growing around our coral colonies to measure increases in nutrient concentrations [80]. This method may provide more accurate measures of nutrient concentrations, which are difficult to detect in oligotrophic waters.

### 4.3. Shared ASVs Indicate Microbial Transmission Amongst Members of This Trophic Symbiosis

Although we did not directly test the transmission of microbes across the three hosts, examining the shared microbes between the hosts gives us insights into which microbes are potentially being transmitted. The most abundant *Endozoicomonas* ASV (Figure S4, Endo_Seq1, Endo_Seq2, for sequence see File S4), is the only *Endozoicomonas* ASV shared among all three hosts (Figure 6, *Endozoicomonas*_1, *Endozoicomonas*_2). Its high abundance in corals may lead to its transfer to the algal matrix and then algae to fish, and/or coral directly to fish through consumption during grazing of the coral for algal matrix farming. However, fish and algal microbiomes also contain other *Endozoicomonas* ASVs with lower abundances and this makes it hard to infer which host is transmitting which *Endozoicomonas* strain, or whether each strain is endemic to each host. In addition, we expected to see more bacteria shared between fish and algal matrix samples since *S. nigricans* farm and exclusively eat the turf algal matrix. However, algal matrix microbiomes were the most diverse but only shared about 6% of their bacteria with fish microbiomes, and only 0.5% of their bacteria with coral microbiomes.

From the ASVs that were more abundant in both fish and algal matrix microbiomes, ASVs within the Family *Vibrionacae* are of interest because they have been shown to proliferate and be pathogenic to corals by forming tissue lesions that affect coral-algal symbiont photosynthetic efficiency (*Vibrio coralliilyticus*) [81,82,83,84]. In addition, *Ruegeria* was more abundant in fish and algal matrix microbiomes, and certain strains of this bacteria can inhibit growth of *Vibrio* species in corals or may be opportunistic pathogens [85]. It is unclear whether these ASVs are being transmitted from either or both the algal matrix and fish to the coral, but their higher abundance in fish and algal matrix microbiomes indicates these hosts as potential vectors of these coral pathogens [26,27,81]. 

## 5. Conclusions

Our study is first to examine the microbiomes of three closely-associated hosts in the coral reef ecosystem under one experimental nutrient-pulse period. We found that the three host, *Porites lobata*, *Stegastes nigricans*, and its farmed algal matrix, microbiomes respond uniquely to changes across time and/or treatment. The most striking difference between the host microbiomes and their response to nutrient enrichment over time was the diversity and evenness of the microbial communities. Fish and algal matrix microbiomes were more diverse and even than coral microbiomes. This variability may have contributed to fish and algal microbiome resistance to change under nutrient enrichment alone, but does not explain the significant changes we see over time or the treatment*time interaction. Since we only manipulated one environmental variable, it is unclear whether other environmental factors were contributing to these changes or compounding the effect of nutrient enrichment over time. This indicates that while increased microbial diversity may prevent major changes in response to one stressor, such as nutrient pollution, it does not guarantee a stabilized microbiome across time. Conversely coral microbiomes did not change significantly over time, which may be due to their highly uneven microbiome dominated by one genus *Endozoicomonas*, suggesting it provides a mechanism of stability. However, nutrient enrichment alone had a significant effect on coral microbiome richness and dispersion. The more noticeable effect of nutrient enrichment on coral microbiomes may have to do with the susceptibility of change in the coral-algal symbiosis under nutrient stress [35,36,37,86,87] which leads to a marked change in the overall coral microbiome and coral physiology [88,89]. 

Our experiment has shown that although these three organisms have tightly linked trophic interactions, their microbiomes tell a different story. While corals provide a structure for algae to grow and for fish to find shelter in, their microbiomes remain unique but susceptible to change under nutrient enrichment. Because the fish actively eat and farm the turf algae, they have a more direct contact with one another, yet their microbiomes are dissimilar and respond differently to temporal changes, in ways that are not intuitively expected. Our study does show that under a stressor such as nutrient enrichment, host microbiomes will respond differently regardless of their proximity or ecological role in a trophic dynamic. In other words, the microbial communities of these hosts do not exactly mirror their macro-scale interactions as the microbiomes respond to nutrient pollution in more complex ways. 

The ability to examine each host microbiome under a single experiment also allowed us to uncover which bacteria were shared among the three hosts. The 51 shared ASVs give us the first insights into how linked the microbiomes of these coral reef organisms are. While we were unable to specify the role of mechanism behind bacterial sharing, the number of ASVs shared between the physically linked microbiomes reflected trophic dynamics, (i.e., fish, which directly touch/feed on the and algal matrix shared more ASVs with the algal matrix than with coral microbiomes) while also highlighting how unique each microbiome is despite the interconnectedness of these hosts. More work needs to be done to understand the mechanisms and pathways of microbial transmission among these organisms as well to understand the functional nature of these relationships. Nevertheless, our study shows that microbiomes provide innovative insights into how anthropogenic stressors are impacting vulnerable but essential marine ecosystem members via their smallest members—the microbes.

## Data Availability

Raw read data can be found at NCBI SRA, BioProject ID: PRJNA752131, which will be made public once the manuscript is published.

## Supplementary Materials

The following are available online at https://www.mdpi.com/article/10.3390/microorganisms9091873/s1, Figure S1: Histogram of the number of read counts within algal matrix microbiome samples, Figure S2: Water nutrient concentrations over time, Figure S3: Community structures of the ASVs found in each host, Figure S4: Relative abundance of coral microbiome *Endozoicomonas* ASVs across time, Figure S5: Changes in coral *Endozoicomonas* within sample diversity, Figure S6: Changes in algal matrix *Salinirepens* and fish *Marinomonas* abundance compared to changes in rainfall across time, Table S1: Changes in taxa numbers throughout data processing pipeline, Table S2: Effects of treatment, time, and treatment*time interaction on microbial community alpha diversity metrics, Table S3: Effects of treatment, time, and treatment*time interaction on microbial community dissimilarity, Table S4: Effects of treatment, time, and treatment*time interaction on microbial community group dispersion, Table S5: BLASTn similarities (top 5 based on Max Score) of differentially abundant genera identified within Family *Vibrionaceae* in fish microbiome samples, File S1: coral_read_nums.txt, File S2: algae_read_nums.txt, File S3: fish_read_nums.txt, File S4: Coral_Endo_ASV_seq.txt.
