# Supplementary material for: Nutrient Enrichment Predominantly Affects Low Diversity Microbiomes in a Marine Trophic Symbiosis between Algal Farming Fish and Corals
Source: Microorganisms. 2021 Sep 3;9(9):1873. doi: 10.3390/microorganisms9091873 (PMC8471015; doi:10.3390/microorganisms9091873)
Supplement: Supplementary file 1 [file microorganisms-09-01873-s001.zip › microorganisms-1356818-supplementary.pdf]

Supplemental Figures

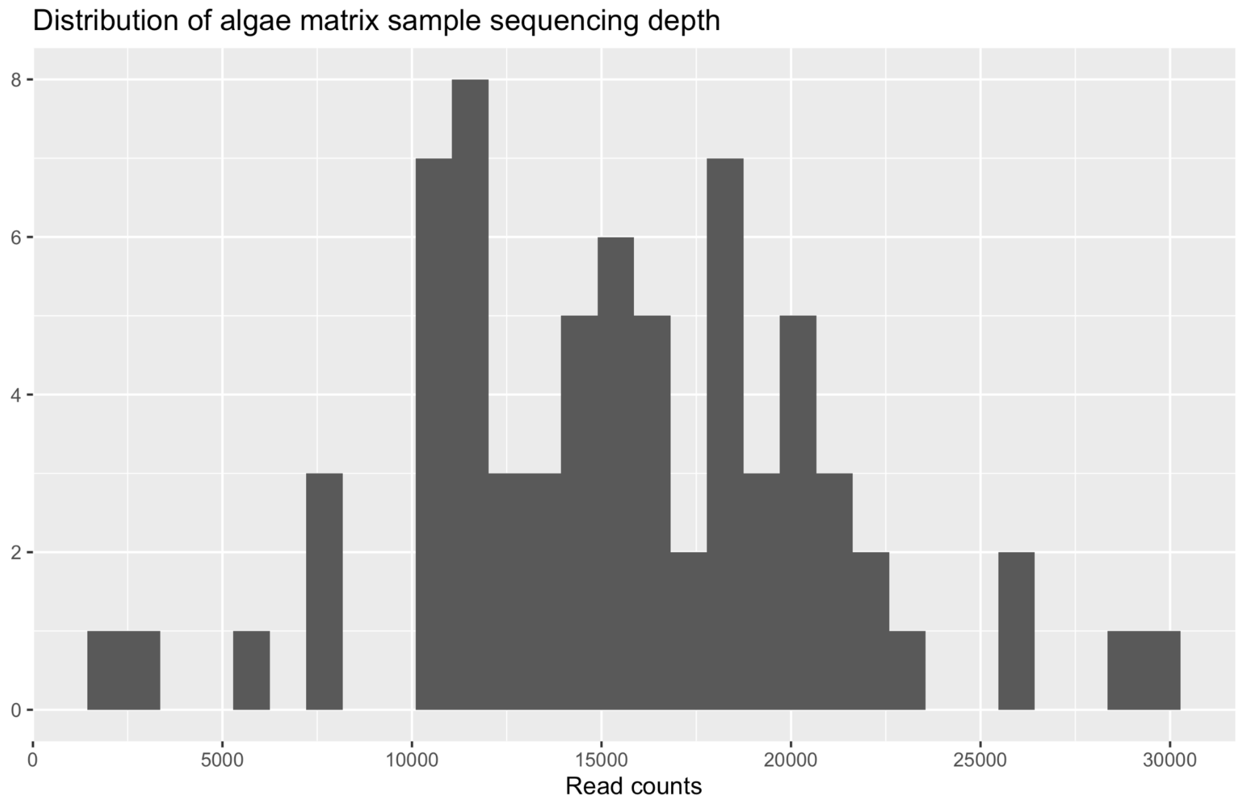

Figure S1: Histogram of the number of read counts within algal matrix microbiome samples. Samples with low read counts (<5,000) were discarded resulting in two samples (represented by the left-most box) being removed from further analysis.

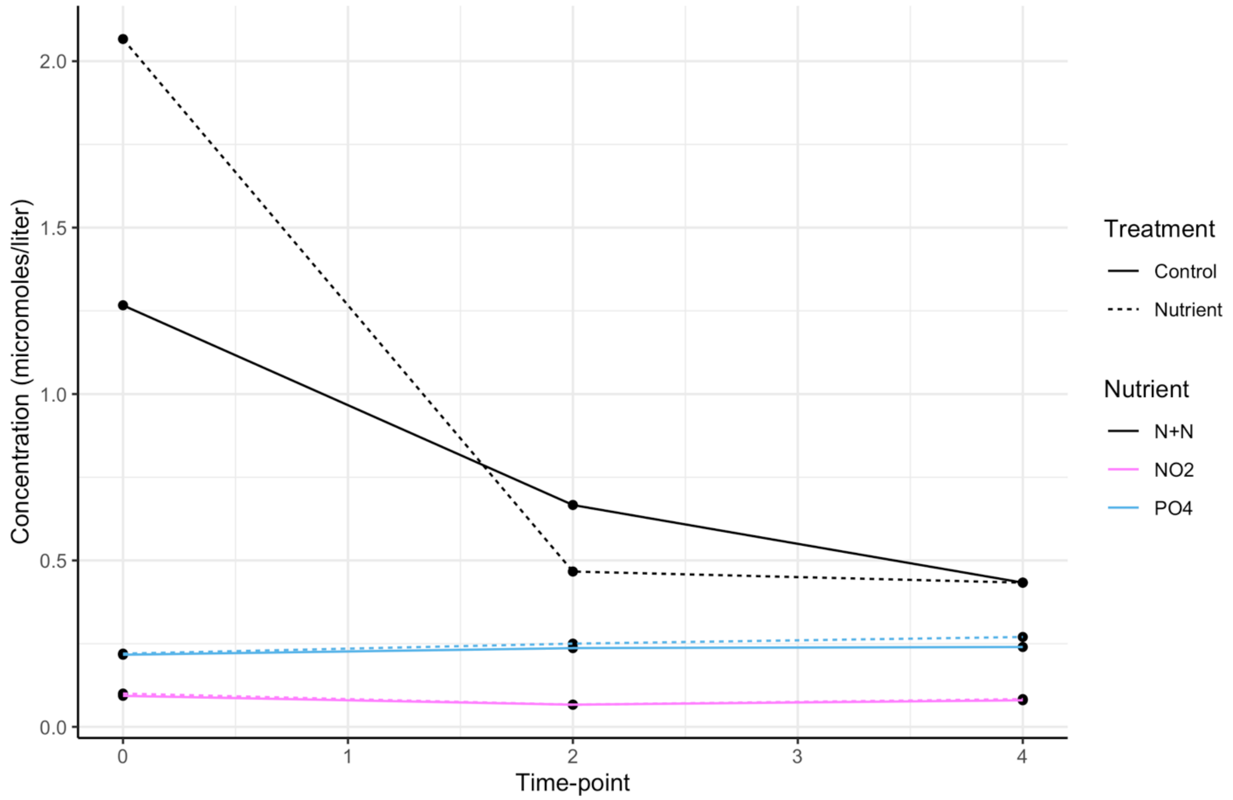

Figure S2: Water nutrient concentrations over time. Concentrations of phosphate (PO4), nitrate and nitrite (N+N), and nitrite (NO2) across the three time-points (T0, T2, and T4) water samples were taken.

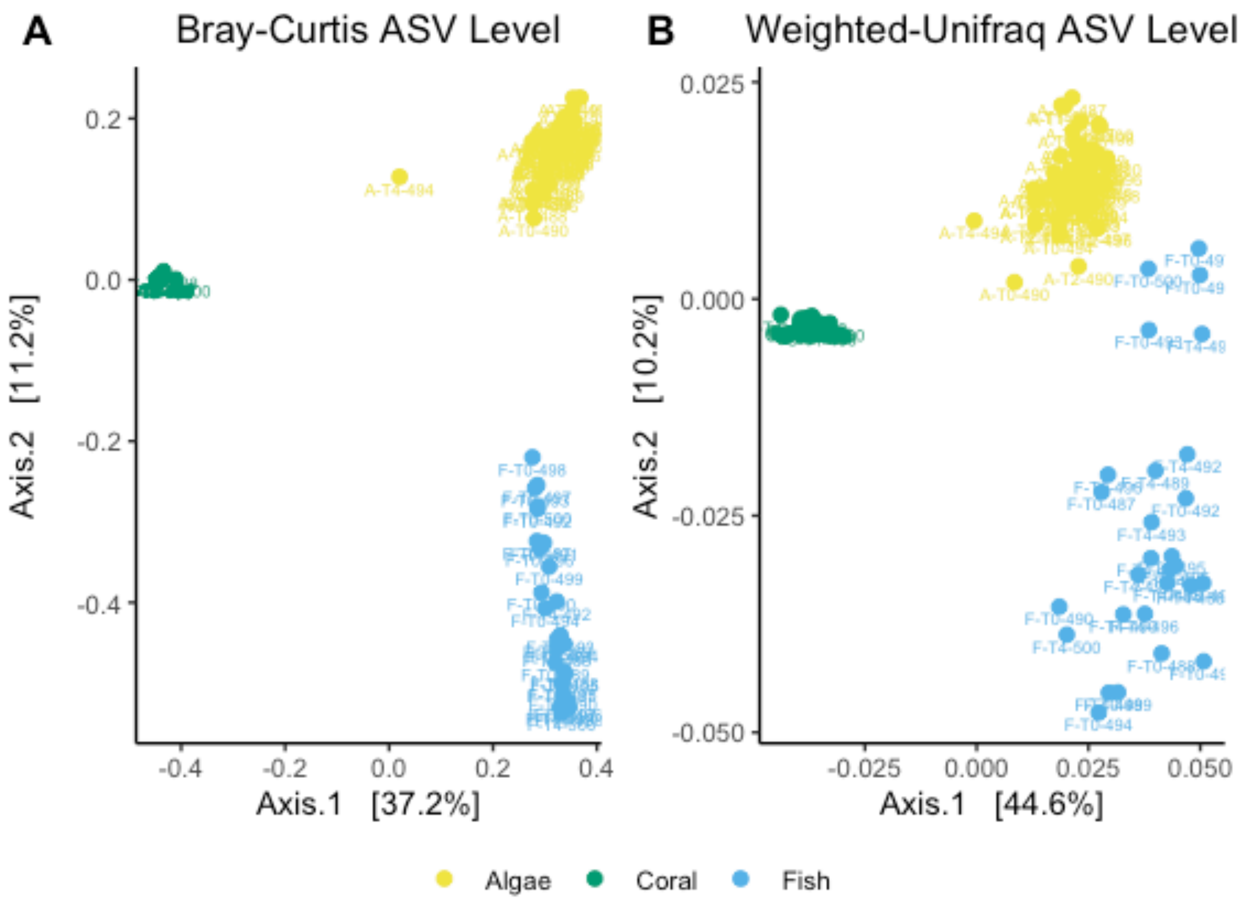

Figure S3: Community structures of the ASVs found in each host. A) Bray-curtis dissimilarity measures of ASVs. B) Weighted-unifraq distance measures of ASVs. Point colors indicate host. The farther points are from one another the more dissimilar their ASV communities are.

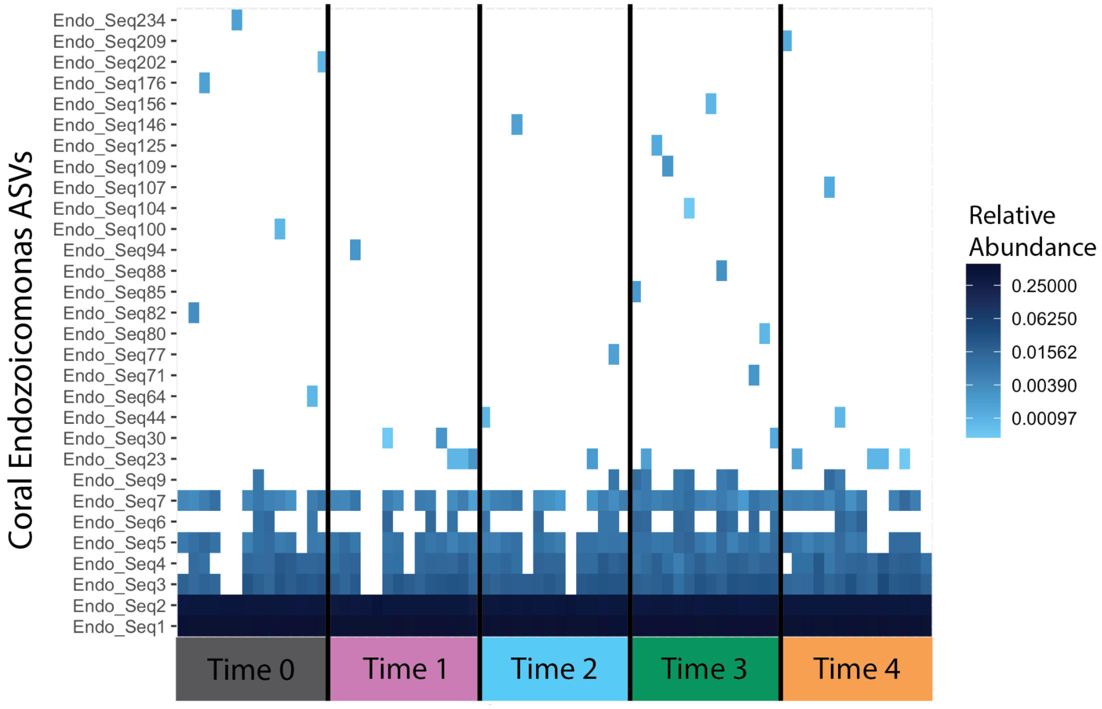

Figure S4: Relative abundance of coral microbiome *Endozoicomonas* ASVs across time. Darker colors indicate higher abundance, and no color indicates an absence of that taxa.

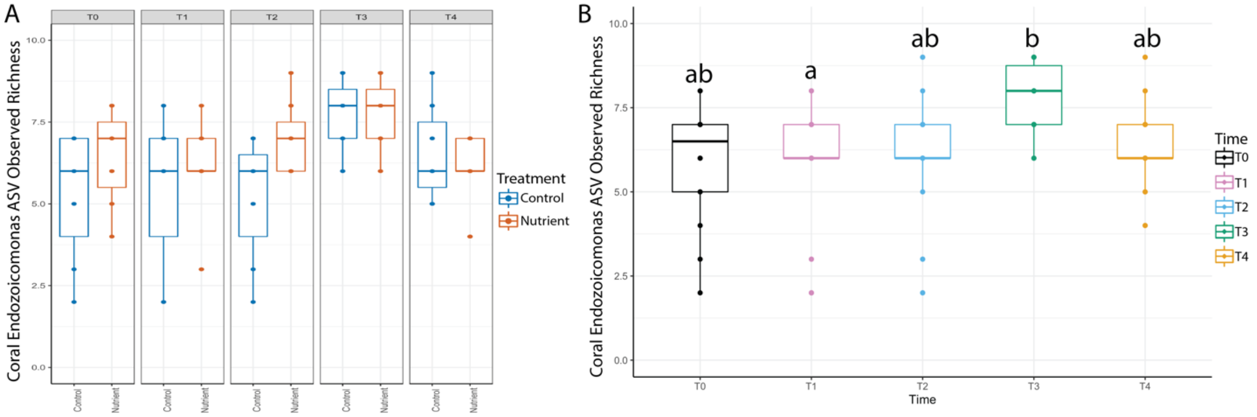

Figure S5: Changes in coral *Endozoicomonas* within sample diversity. A) Changes in coral *Endozoicomonas* across time and treatment show similar levels of richness between treatment for T0, T1, T3, and T4. T2 shows that largest difference between control and nutrient treated corals however this difference was not statistically significant. Across time, T3 had the highest increase in diversity, which was deemed statistically significant. B) Significant changes in coral *Endozoicomonas* observed richness across time, with a significant increase occurring between T1 and T2.

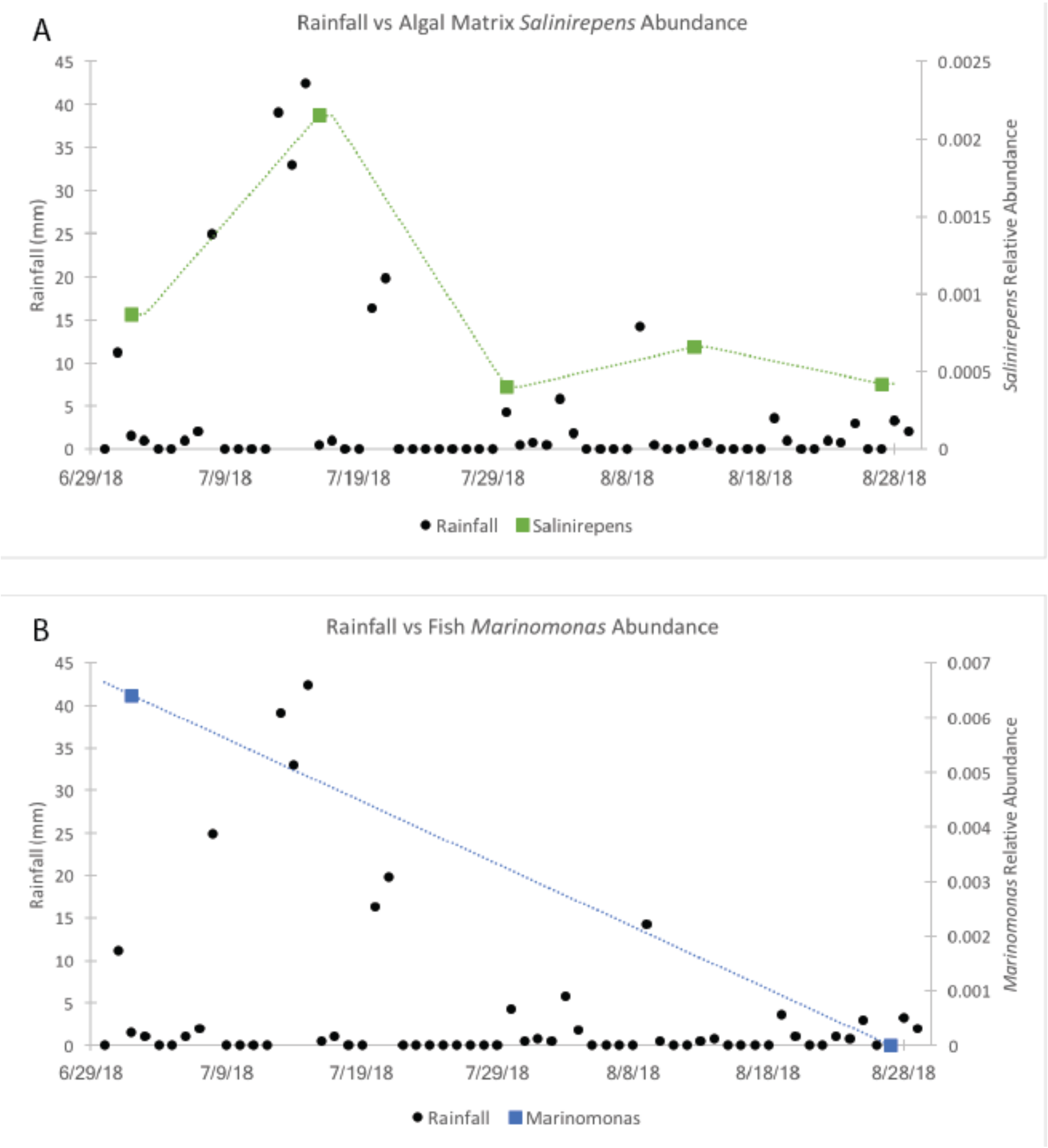

Figure S6: Changes in algal matrix *Salinirepens* and fish *Marinomonas* abundance compared to changes in rainfall across time. Each square point represents A) *Salinirepens* (green squares) and B) *Marinomonas* (blue squares) average abundance across both treatments for each sampled time point. A) *Salinirepens* abundance within algal matrix microbiomes follows rainfall patterns across all five sampled timepoints. B) *Marinomonas*, a common marine microbe, abundance decreases to 0 across the two sampled timepoints.

Supplemental Tables

Table S1: Changes in taxa numbers throughout data processing pipeline.

	**Number of taxa (ntaxa) for each host**		
**Data Processing Step**	ntaxa Coral	ntaxa Fish	ntaxa Algae
Silva (after DADA2)	732	3,893	22,791
Mitochondria, Cholorplast, NA filtering	578	3,457	21,112
Genus tip_glom	335	1,117	6,742
Rarefy	215	1,095	6,179

Table S2. Effects of treatment, time, and treatment*time interaction on microbial community alpha diversity metrics. Alpha diversity metrics (Observed Richness, Simpson's index) were regressed against experimental variables using LMMs with experimental variables (treatment, time, treatment*time) as the fixed effect and coral colony as random effects. Observed richness was log-transformed for each host, while Simpson's Index was arcsine-transformed to improve normality only for coral samples. P-values were approximated with the Type III Analysis of Variance with Satterthwaite's method.

Coral Samples

**Alpha diversity measure**	**Variables**	**Sum Sq**	**Mean Sq**	**NumDF**	**DenDF**	**F value**	**P value**
**Observed Richness**	Treatment	5.0768	5.0768	1	12.002	4.9885	**0.04532**
	Time	8.7406	2.1851	4	48.003	2.1471	0.08934
	Treatment*Time	6.4114	1.6028	4	48.003	1.5750	0.19616
**Simpson's diversity**	Treatment	0.094118	0.094118	1	60	7.6655	**0.007477**
	Time	0.027048	0.006762	4	60	0.5507	0.699194
	Treatment*Time	0.055358	0.013840	4	60	1.1272	0.352293

Algal Matrix Samples

**Alpha diversity measure**	**Variables**	**Sum Sq**	**Mean Sq**	**NumDF**	**DenDF**	**F value**	**P value**
**Observed Richness**	Treatment	2.776	2.776	1	11.961	0.7571	0.40138
	Time	154.404	38.601	4	46.487	10.5282	**3.718e-6**
	Treatment*Time	35.770	8.942	4	46.487	2.4390	0.06007
**Simpson's diversity**	Treatment	4.316e-5	4.3158e-5	1	11.306	0.2018	0.6617
	Time	1.1743e-3	2.9357e-4	4	45.837	1.3729	0.2581

	Treatment*Time	9.0243e-4	2.2561e-4	4	45.837	1.0551	0.3895

Fish Samples							
**Alpha diversity measure**	**Variables**	**Sum Sq**	**Mean Sq**	**NumDF**	**DenDF**	**F value**	**P value**
**Observed Richness**	Treatment	14.6557	14.6557	1	12	3.0223	0.1077
	Time	4.8053	4.8053	1	12	0.9909	0.3392
	Treatment*Time	13.4057	13.4057	1	12	2.7645	0.1223
**Simpson's diversity**	Treatment	7.431e-4	7.431e-4	1	12	0.8167	0.38392
	Time	1.143e-4	1.143e-4	1	12	0.1257	0.72912
	Treatment*Time	8.666e-3	8.666e-3	1	12	9.5242	**0.00943**

Note: p-values defined as significant at a threshold of 0.05 are highlighted in bold.

Table S3. Effects of treatment, time, and treatment*time interaction on microbial community dissimilarity. PERMANOVA results for differences in community dissimilarity measured by Bray Curtis dissimilarity measures by experimental variable. Only coral samples were log-transformed.

Coral Samples							
**Dissimilarity Measure**	**Measurements**	**Df**	**SumsOfSqs**	**MeanSqs**	**F.Model**	**R^2**	***P***
**Bray Curtis**	Treatment	1	0.3201	0.32011	2.9362	0.04086	**0.006**
	Time	4	0.5326	0.13314	1.2213	0.06798	0.152
	Treatment*Time	4	0.4401	0.11002	1.0092	0.05618	0.435
	Residuals	60	6.5411	0.10902		0.83498	
	Total	69	7.8339			1.00000	

Algal Matrix Samples							
**Dissimilarity Measure**	**Measurements**	**Df**	**SumsOfSqs**	**MeanSqs**	**F.Model**	**R^2**	***P***
**Bray Curtis**	Treatment	1	0.2347	0.23467	1.29354	0.01894	0.071
	Time	4	0.9817	0.24542	1.35281	0.07924	**0.008**

	Treatment*Time	4	0.6499	0.16247	0.89559	0.05246	0.859
	Residuals	58	10.5219	0.18141		0.84935	
	Total	67	12.3881			1.00000	

Fish Samples

Dissimilarity Measure	Measurements	Df	SumsOfSqs	MeanSqs	F.Model	R²	*P*
**Bray Curtis**	Treatment	1	0.0728	0.07282	0.5100	0.01494	0.923
	Time	1	1.0390	1.03901	7.2762	0.21321	**0.001**
	Treatment*Time	1	0.3343	0.33434	2.3414	0.06861	**0.025**
	Residuals	24	3.4271	0.14280		0.70325	
	Total	27	4.8733			1.00000	

Note: p-values defined as significant at a threshold of 0.05 are highlighted in bold.

Table S4. Effects of treatment, time, and treatment*time interaction on microbial community group dispersion. Results of PERMDISP tests for differences in group dispersion for all 3 hosts with Bray Curtis dissimilarity measurements for treatment, time, and treatment*time.

Coral Samples

Dissimilarity Measure	Variable	Df	Sum Sq	Mean Sq	F value	P
**Bray Curtis**	Treatment	1	0.14342	0.143415	5.3722	**0.02348**
	Time	4	0.09777	0.024442	0.901	0.4687
	Treatment*Time	9	0.41346	0.04594	1.9675	0.05919

Algal Matrix Samples

Dissimilarity Measure	Variable	Df	Sum Sq	Mean Sq	F value	P
**Bray Curtis**	Treatment	1	0.002647	0.002647	1.112	0.2955
	Time	4	0.042304	0.0105761	4.553	**0.002708**
	Treatment*Time	9	0.062803	0.0069781	2.8941	**0.006777**

Fish Samples

Dissimilarity Measure	Variable	Df	Sum Sq	Mean Sq	F value	P
**Bray Curtis**	Treatment	1	0.00089	0.0008853	0.0525	0.8205
	Time	1	0.15678	0.156783	23.562	**4.939e-05**
	Treatment*Time	3	0.20402	0.068007	13.991	1.764e-05

Note: p-values defined as significant at a threshold of 0.05 are highlighted in bold.

Table S5: BLASTn similarities (top 5 based on Max Score) of differentially abundant genera identified within Family *Vibrionaceae* in fish microbiome samples.

Description	Scientific Name	Max Score	Total Score	Query Cover	E value	Per. ident	Acc. Len	Accession
Vibrio sinaloensis strain W37 16S ribosomal RNA gene, partial sequence	*Vibrio sinaloensis*	401	401	99%	1.00E-107	94.59	1332	MT605274.1
Bacterium strain MRB-09 16S ribosomal RNA gene, partial sequence	bacterium	401	401	99%	1.00E-107	94.59	1414	KX941474.1
Vibrio sp. strain B-RD_1 16S ribosomal RNA gene, partial sequence	*Vibrio sp.*	401	401	99%	1.00E-107	94.59	1042	MF033528.1
Vibrio sinaloensis partial 16S rRNA gene, strain JC96, isolate Pkl-8	*Vibrio sinaloensis*	401	401	99%	1.00E-107	94.59	1348	FR837600.1
Vibrio vulnificus strain G23-21 16S ribosomal RNA gene, partial sequence	*Vibrio vulnificus*	401	401	99%	1.00E-107	94.59	1083	GU143892.1
